# Exploring the mediating roles of sport commitment and resilience between life satisfaction and social anxiety among Chinese primary school students

**DOI:** 10.3389/fpsyg.2025.1619817

**Published:** 2025-11-05

**Authors:** Wencong Kan, Menglin Xu, Yufen Wang, Changhui Zuo, Lei Yi, Xiaoxi Dong

**Affiliations:** ^1^Department of Sports Teaching and Research, Lanzhou University, Lanzhou, Gansu, China; ^2^Boston Medical Center, Boston, MA, United States; ^3^Nanjing Cuipingshan Primary School, Nanjing, Jiangsu, China; ^4^Faculty of Sports and Health, Tianjin University of Traditional Chinese Medicine, Tianjin, China; ^5^Graduate School, Guangzhou Sport University, Guangzhou, Guangdong, China

**Keywords:** life satisfaction, social anxiety, sport commitment, personal resilience, caregiver resilience, primary school students

## Abstract

**Background:**

The mental health of children and adolescents has become urgent global concerns, particularly in China, where intense academic competition, societal pressures, and fast-paced lifestyles exacerbate psychological challenges. These factors significantly hinder healthy psychological development, underscoring the need for innovative approaches to address youth subjective wellbeing. This study investigates the mediating roles of resilience and sport commitment in the relationship between social anxiety and life satisfaction.

**Methods:**

A cross-sectional design was employed, involving 948 primary school students (446 females, 502 males) from four major Chinese cities. Participants completed an online survey assessing sport commitment, life satisfaction, resilience (personal and caregiver-related), and social anxiety. Path analysis using a maximum likelihood estimator was conducted to examine the mediating roles of sport commitment, personal resilience, and caregiver resilience in the relationship between social anxiety and life satisfaction. Indirect effects were evaluated using bootstrap sampling with a bias-corrected 95% confidence interval.

**Results:**

The findings revealed a satisfactory model fit, χ2(5) = 8.414, *p* = 0.135, CFI = 0.998, TLI = 0.992, RMSEA = 0.027, 90% CI = (0.000, 0.057), and SRMR = 0.018, with 36.4% of the variation in life satisfaction that could be explained by the model. Specifically, sport commitment, personal resilience, and caregiver resilience significantly mediated the relationship between social anxiety and life satisfaction. The total indirect effect was significant, accounting for 67.93% of the total effect. In addition, social anxiety was negatively associated with life satisfaction.

**Conclusion:**

This study advances the literature by highlighting the protective roles of resilience and sport commitment in mitigating the impact of social anxiety on life satisfaction. It underscores the importance of fostering resilience at individual, familial, and societal levels and emphasizes the role of educational systems in promoting youth subjective well-being. Practical implications include integrating sport-based activities and resilience-building programs into school curricula, as well as fostering collaboration among parents, educators, and community stakeholders. Future research should explore targeted intervention strategies and their long-term impacts on youth subjective wellbeing.

## 1 Introduction

Mental disorders have consistently ranked among the top ten causes of global burden, with no signs of decline since 1990 (GBD 2019 Mental Disorders Collaborators, 2022). Although there is no universally accepted definition of mental disorder, anxiety is a key contributor to declining individual mental health (Biddle et al., 2019). For instance, anxiety disorders increased from 194.9 million to 301.4 million cases globally between 1990 and 2019 (GBD 2019 Mental Disorders Collaborators, 2022). This rising prevalence of anxiety poses a significant threat to global public health and decreases individuals’ life satisfaction (Luengo-González et al., 2023). Specifically, Chinese children and adolescents are a vulnerable population at a higher risk of anxiety due to significant pressures from intense competition for future opportunities and the demands of maintaining a fast-paced lifestyle in contemporary society (Guo and Liang, 2023). Given the concern regarding the negative relationship between anxiety disorders and life satisfaction, it is crucial to ascertain protective health factors to mitigate the harmful effects of anxiety disorders on both individual and public health.

Social anxiety, a type of anxiety disorder (WHO, 2023), significantly impairs daily functioning and quality of life (Bogels et al., 2010). This impairment may be because social relationships play a fundamental role in emotional development (La Greca et al., 1988). Social anxiety is characterized by excessive fear of negative evaluation in social situations, often leading to feelings of humiliation, embarrassment, or rejection (Kogan et al., 2016). Previous studies have indicated that social anxiety is associated with increased negative factors such as bullying victimization (Wu et al., 2021), internet addiction (Ye et al., 2021), suicidal ideation (Tan et al., 2024), and Loneliness (Lin and Fan, 2023), and decreased positive factors such as perceiving support from family, friends, and others (Ren and Li, 2020), physical activity (Wang H. et al., 2024), social cognitive ability (Pearcey et al., 2021), and resilience (Jefferies et al., 2021). Moreover, social anxiety frequently manifests during childhood and adolescence, with a significant increase in incidence between the ages of 9 and 19 years (Beesdo et al., 2007). Although a meta-analysis indicated that the pooled prevalence of social anxiety disorder among Chinese children, adolescents, and young adults was estimated to be 2.1%, the study reported that the proportion of study participants experiencing social anxiety symptoms was estimated to be 23.5% (Tang et al., 2022). This high prevalence of social anxiety symptoms highlights the need for targeted mental health prevention among Chinese children and adolescents. In addition, primary school students represent a particularly critical subgroup within this population. At this developmental stage, children’s social cognition is still maturing, and higher levels of social anxiety have been associated with difficulties in specific aspects of social cognition (Pearcey et al., 2021). Consequently, social anxiety at this age may exert stronger and more enduring effects on resilience, sport commitment, and life satisfaction than at later stages of development.

Life satisfaction is a crucial component of subjective wellbeing (Diener et al., 1999). It involves a cognitive process of evaluative judgments, influenced not only by comparing one’s present situation to personal standards but also by a range of life experiences (Diener et al., 1985). Thus, life satisfaction can be regarded as the attainment of benefits that enhance an individual’s personal hedonic satisfaction (Çikrikci et al., 2022). This perspective highlights the importance of personal life experiences in shaping satisfaction, which is influenced by various events and environments. Although the process of adapting to events generally stabilizes life satisfaction, certain life events and experiences in specific domains can have lasting impacts, such as stressful experiences (Pavot and Diener, 2008). Research indicates that unpleasant experiences, including anxiety, may disproportionately impact an individual’s life satisfaction compared to positive experiences, with undesirable events potentially affecting subjective wellbeing up to five times more intensely than pleasant events (Hanson and Mendius, 2009). Moreover, previous studies have indicated that life satisfaction is negatively associated with perceived stress (Zheng et al., 2019), depression (Wang et al., 2009), and social anxiety (Wang and Yao, 2020), while a positive association has been reported between life satisfaction and physical activity (Dong et al., 2023a) as well as resilience (Shi et al., 2015). The present study aims to explore how resilience and sport commitment mediate the relationship between social anxiety and life satisfaction, suggesting that these mediators may be associated with the negative factor of social anxiety and life satisfaction.

Resilience plays a crucial role in protecting adolescents’ wellbeing, and there is growing academic interest in investigating its impact as a protective factor (Hjemdal et al., 2006). Resilience is defined as the dynamic process of efficiently handling significant stress or trauma and the capacity for resilience to recover is facilitated by a combination of personal and environmental resources, with its experience varying across different stages of life (Windle, 2011). Studies have demonstrated that personal resilience contributes to positive outcomes such as subjective wellbeing (Chuning et al., 2024) and sleep quality (Ashour et al., 2024). In addition to these positive outcomes, personal resilience also mitigates the negative impacts of internalizing issues such as anxiety and depression (Şanlı et al., 2024) and childhood trauma (Hu et al., 2024), as well as externalizing issues such as cell phone addiction (Zhang and Gao, 2023). For instance, adolescents with higher levels of resilience exhibit fewer behavioral and emotional problems despite experiencing psychological maltreatment (Arslan, 2016). Therefore, personal resilience may act as a mediator, alleviating the impact of risk factors that harm health while enhancing the positive effects that improve subjective wellbeing.

Resilience involves multisystemic processes that improve wellbeing through interactions across psychological, social, and environmental systems (Ungar and Theron, 2020). A key aspect of resilience is the ability of individuals to access supportive resources in meaningful ways, such as those provided by families and communities, to foster wellbeing (Resilience Research Centre, 2018). From this perspective, the capacity to overcome adverse experiences and utilize relationships with parents are two key factors in maintaining wellness (Liebenberg et al., 2012). Indeed, primary caregiver parents serve as a crucial support system, helping children navigate adverse situations and fostering their growth and development (Zhang Y. et al., 2024). This highlights the importance of parents creating a harmonious and stable nurturing environment, essential for a child’s healthy growth and development (Qi and Yang, 2024). Previous studies have shown a positive relationship between family resilience and personal resilience (Qi and Yang, 2024; Wang Y. et al., 2024; Zhang Y. et al., 2024). This emphasizes that personal resilience and parent resilience interact and interrelate in cultivating subjective wellbeing. Rather than solely studying personal resilience, investigating the relationships between personal resilience and parent resilience in mediating negative effects and positive factors of subjective wellbeing may provide valuable insights into the field of mental health. We hypothesize that the personal resilience and parent resilience may be associated with life satisfaction and social anxiety.

One of the most promising factors to increase adolescents’ subjective wellbeing is physical activity. Previous studies have confirmed a positive relationship between physical activity and wellbeing (McMahon et al., 2017), and a negative relationship between physical activity and mental disorders such as anxiety and depression (Gedda-Muñoz et al., 2023). Furthermore, physical activity also has a strong reciprocal relationship with resilience. For example, physical activity is not only revealed as a positive factor correlated with resilience (Kamini, 2019) but also as a causal variable contributing to resilience among adolescents (Guo and Liang, 2023). Thus, the benefits of physical activity in enhancing resilience are partially attributed to its capacity to cultivate positive physiological and psychological development by minimizing the negative impact of adverse experiences on health (Arida and Teixeira-Machado, 2021). Moreover, physical activity and resilience are interrelated and mutually beneficial, improving subjective wellbeing, reducing the effects of mental disorders, and offering numerous health benefits for children and adolescents. However, despite these benefits, a high rate of insufficient physical activity persists among adolescents globally. Specifically, data from 146 countries indicate that approximately 81% of adolescents aged 11–17 do not achieve sufficient levels of physical activity (Guthold et al., 2020). The high rate of physical inactivity among adolescents has put individual and public healthcare systems at risk (Ding et al., 2016; Lee et al., 2012). Therefore, it is urgent to increase participation levels in physical activity.

Sport Commitment Theory, a key concept in motivational psychology, provides valuable insights into the mechanisms by which physical activity influences subjective wellbeing. This theory defines sport commitment as a psychological construct that embodies an individual’s motivation and resolve to continue engaging in sport (Scanlan et al., 1993a). This construct encompasses sport enjoyment, involvement alternatives, personal investments, social constraints, and involvement opportunities, (Carpenter et al., 1993; Scanlan et al., 1993a). Grounded in examining motivations for persistent participation in sports, this theoretical model illustrates how attachment, as a component of motivational force, drives persistent involvement and fosters a comprehensive understanding of commitment (Carpenter et al., 1993; Scanlan et al., 1993b). Moreover, a positive association exists between sport commitment and satisfaction, with sport enjoyment particularly emphasizing the positive affective response to sport experiences (Carpenter and Scanlan, 1998). Empirical evidence supports these theoretical claims. For instance, a study demonstrated that sport commitment is positively associated with the pleasure factor of participation motivation and continuous participation intention and also mediates the relationship between the pleasure factor of participation motivation and continuous participation intention (Choi, 2023). This underscores the importance of emotional experiences in fostering long-term commitment, highlighting how collective emotional experiences contribute to satisfaction and sustained engagement in physical and sports activities (Hall et al., 2017). Understanding the associations between these emotional dynamics and motivational forces is crucial for cultivating sport commitment and maintaining long-term engagement in physical activity. According to Hodge et al. (2023), the connection between sport commitment and Self-Determination Theory serves as a key framework for predicting behavioral persistence and continued participation in sports. Previous studies have shown a positive relationship between sport commitment and intrinsic motivation (Zahariadis et al., 2006), autonomous motivation and need satisfaction (Murillo et al., 2022), subjective exercise experience and exercise adherence (Tian and Shi, 2022), satisfaction (Wilson et al., 2004), and exercise behavior (Zhang Z. et al., 2024). Therefore, Sport Commitment Theory offers a theoretical foundation for understanding how commitment to physical activity can serve as a mediator in the relationship between social anxiety and life satisfaction.

**FIGURE 1:**
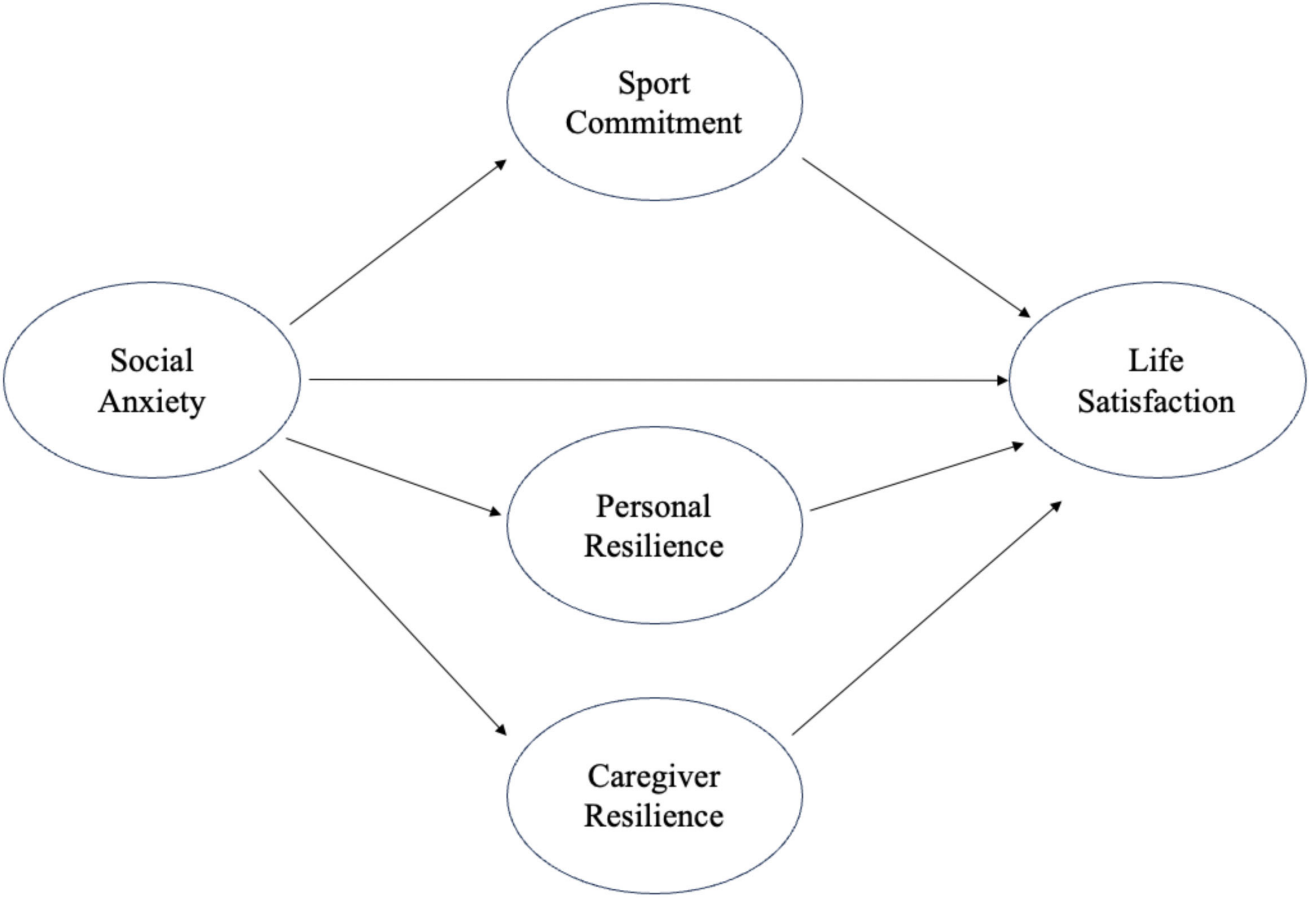
Hypothesized conceptual model.

Building on the integrated theoretical perspectives, this study aims to examine the mediating roles of resilience and sport commitment in the relationship between social anxiety and life satisfaction. The inclusion of both resilience and sport commitment as mediators is grounded on the theoretical understanding that these constructs interact to the negative effects of social anxiety, thereby fostering life satisfaction. This research seeks to uncover the mechanisms through which these constructs influence subjective wellbeing, providing valuable insights for future interventions targeting mental health and physical activity engagement in children and adolescents. Based on the theoretical frameworks of sport commitment and resilience, this study addresses two research questions (RQ) and proposes four hypotheses. Additionally, the hypothesized conceptual model is presented in Figure 1.

RQ1: What are the relationships between sports commitment, resilience, social anxiety, and life satisfaction?

RQ2: Do sport commitment and resilience mediate the relationship between life satisfaction and social anxiety?

H1: Sport commitment, resilience, and life satisfaction are positively associated.

H2: Social anxiety is negatively associated with sport commitment, resilience, and life satisfaction.

H3: Resilience mediates the relationship between life satisfaction and social anxiety.

H4: Sport commitment mediates the relationship between life satisfaction and social anxiety.

## 2 Materials and methods

### 2.1 Participants and procedure

This study employed a cross-sectional design with a convenience sampling technique to collect research data. After receiving approval from the Ethics Committee of the University (reference number: 2023013, data approved: 2023.02.22), researchers contacted potential gatekeepers working in primary schools in China to facilitate participant recruitment. Four of these gatekeepers agreed to assist by sharing the researchers’ online survey link with their students’ parents. The online survey directed participants to the study instrument, which consisted of two sections: a Parental Informed Consent form and the survey itself. Once respondents’ parents signed the Parental Informed Consent form, the survey was provided to their children, who then completed it. Parental Informed Consent forms were obtained from all participants’ parents. Participants were recruited from four primary schools in four Chinese cities: Beijing, Nanjing, Shijiazhuang, and Handan. The sample comprised 948 primary school students in China, including 446 female students and 502 male students, and the mean age was 10.17 years (SD = 1.69). Moreover, the distribution of participants was as follows: 154 in grade 1, 8 in grade 2, 196 in grade 3, 133 in grade 4, 246 in grade 5, and 211 in grade 6.

### 2.2 Measures

#### 2.2.1 Sport commitment

The Sport Commitment Scale (SCS) was developed by Chen and Li (2006). It is based on the sport commitment theory and the sport commitment scale created by Scanlan et al. (1993a). The SCS aims to evaluate and predict Chinese students’ levels of physical activity, and it comprises five factors: sport commitment, sport enjoyment, personal investment, social constraints, and involvement opportunities, with each factor including three items (Chen and Li, 2006). Participants rate each item on a five-point Likert scale ranging from 1 (strongly disagree) to 5 (strongly agree). The SCS demonstrated good reliability and validity within the Chinese population (Chen and Li, 2006; Dong et al., 2024). In this study, the SCS was used to evaluate primary school students’ sport commitment, with a Cronbach’s alpha of 0.89, indicating high internal consistency. Moreover, the composite score was computed by averaging the scores of the individual items.

#### 2.2.2 Life satisfaction

Satisfaction with Life Scale (SWLS, Diener et al., 1985) was used to evaluate primary school students’ life satisfaction in this research. It consists of five items, with responses rated on a seven-point Likert-type scale ranging from 1 (strongly disagree) to 7 (strongly agree). The SWLS is a reliable and valid instrument for evaluating life satisfaction among the Chinese population (Dong et al., 2023a). In this study, the Cronbach’s alpha coefficient was 0.81, indicating high internal consistency. Moreover, the composite score was computed by averaging the scores of the individual items.

#### 2.2.3 Resilience

Child and Youth Resilience Measure-Revised (CYRM-R, Jefferies et al., 2018; Resilience Research Centre, 2018) was used to measure primary school students’ resilience in this study. The CYRM-R consists of two factors: personal resilience and caregiver resilience (Jefferies et al., 2018; Resilience Research Centre, 2018). The personal resilience factor consists of 10 items, and the caregiver resilience factor consists of seven items, with responses rated on a five-point scale: 1 = not at all, 2 = a little, 3 = somewhat, 4 = quite a bit, and 5 = a lot. The personal resilience factor encompasses both intrapersonal and interpersonal elements, and the caregiver resilience factor pertains to characteristics associated with significant relationships with a primary caregiver or family (Jefferies et al., 2018; Resilience Research Centre, 2018). In this study, the Cronbach’s alpha coefficient was 0.90 for personal resilience and 0.84 for caregiver resilience, indicating high internal consistency. Moreover, the composite score was computed by averaging the scores of the individual items.

#### 2.2.4 Social anxiety

The Social Anxiety Scale for Children (SASC), developed by La Greca et al. (1988), is used to evaluate anxiety disorder and serves as an auxiliary tool for assessing feelings of social anxiety in children (Li et al., 2006). Based on the SASC, Li et al. (2006) developed the Chinese version, which consists of 10 items rated on a three-point scale: never, sometimes, and always. In addition, the Chinese version of the SASC includes two factors: fear of negative evaluation (six items) and social avoidance and distress (four items). This scale demonstrates good reliability and validity, with Cronbach’s alpha coefficient of 0.79 for fear of negative evaluation, Cronbach’s alpha coefficient of 0.58 for social avoidance and distress, and Cronbach’s alpha coefficient of 0.79 for the SASC (Li et al., 2006). In this study, the fear of negative evaluation factor, which indicated a good reliability and validity factor of the SASC, was used to measure the feelings of social anxiety of Chinese primary school students, with a Cronbach’s alpha coefficient of 0.86, indicating high internal consistency. Moreover, the composite score was computed by averaging the scores of the individual items.

### 2.3 Statistical analysis

Regarding data screening, since we had a 100% response rate, no missing data approach was needed. Moreover, the original scale formats were used without re-scaling. Normality assumptions were checked by inspecting skewness, kurtosis, and Q-Q plots. Descriptive statistics and Pearson correlations were computed to inspect the characteristics of the variables. Confirmatory factor analysis (CFA) models were used to test the construct validity of each of the key model variables. Discriminant validity was assessed following Fornell–Larcker criterion (Fornell and Larcker, 1981). Subsequently, path analysis within the structural equation modeling was performed to assess the proposed mediation model while controlling for background variables of gender and age. The path model was evaluated using goodness-of-fit indices, including the Comparative Fit Index (CFI), Tucker-Lewis Index (TLI), Root Mean Square Error of Approximation (RMSEA), and Standardized Root Mean Square Residual (SRMR) (Hu and Bentler, 1999). A satisfactory model fit will be determined by CFI, TLI values greater than 0.90, and RMSEA, SRMR values less than 0.08 (Schumacker and Lomax, 2010). The maximum likelihood estimator was used. Further, to test the indirect effects, the bootstrap method with 5,000 bootstrapping samples was adopted, and the significance was tested using a bias-corrected 95% confidence interval. Moreover, common method bias tests were performed for the key model constructs using Harman’s single-factor test (Harman, 1967). Power justification was realized by performing Monte Carlo study (Muthén and Muthén, 2002). In this study, with the final path mediation model as the population model and 1,000 replications, we got > 90% statistical power for all the path coefficient estimation, demonstrating sufficient power. All statistical analyses were conducted using SPSS 26.0 and Mplus 8.0.

## 3 Results

### 3.1 Common method bias

Common method bias was checked using Harman’s single-factor test (Harman, 1967). Principal component analysis showed that the first factor accounted for 31.1% of the variance, below the 40% threshold, suggesting that common method bias was not a serious concern.

### 3.2 Measurement model

CFA results showed that all the five measures embraced decent model fit. In detail, CFI = 0.967, TLI = 0.945, RMSEA = 0.095 (0.078, 0.114), and SRMR = 0.032 for social anxiety; CFI = 0.989, TLI = 0.978, RMSEA = 0.061 (0.037, 0.088), and SRMR = 0.019 for life satisfaction; CFI = 0.953, TLI = 0.939, RMSEA = 0.074 (0.068, 0.081), and SRMR = 0.052 for sport commitment. CFI = 0.943, TLI = 0.926, RMSEA = 0.085 (0.076, 0.095), and SRMR = 0.037 for personal resilience. CFI = 0.969, TLI = 0.949, RMSEA = 0.075 (0.060, 0.091), and SRMR = 0.030 for caregiver resilience. In addition, for each of the five measures, standardized factor loadings were all above 0.50 (for more detailed information, please refer to Supplementary Table 2). These findings showcased that all the measures had satisfactory construct validity.

Then, based on the established measurement models, we calculated McDonald (1999) as a representation of composite reliability. Results showed that composite reliability was 0.86 for social anxiety, 0.81 for life satisfaction, 0.87 for sport commitment, 0.90 for personal resilience, and 0.84 for caregiver resilience. The results showed that the measures embraced good internal consistency.

Further, we performed discriminant validity tests following Fornell–Larcker criterion. Average variance extracted (AVE) values were calculated for each measure, and squared root values of AVE were displayed in the diagonals of correlation table in Table 1. As shown in Table 1, for SASF, SC, and LS, the *sqrt*(*AVE*) values were larger than the correlations with other measures, demonstrating good discriminant validity. For PRS and CRRS, the *sqrt*(*AVE*) values were larger than correlations with SASF, SC, and LS, yet less than r (PRS, CRRS), showing that the resilience scale in general was distinct from other psychological constructs, but had high correlation between its subscales.

**TABLE 1:** Descriptive statistics and Pearson correlations among study variables.

N/A	1	2	3	4	5	6	7
1. Gender (female)	N/A	–	–	–	–	–	–
2. Age	0.02	N/A	–	–	–	–	–
3. SASF	0.06	−0.08*	0.72	–	–	–	–
4. SC	−0.09**	−0.08*	−0.21***	0.57	–	–	–
5. PRS	0.03	0.09**	−0.41***	0.46***	0.69	–	–
6. CRRS	−0.01	−0.01	−0.36***	0.44***	0.78***	0.66	–
7. LS	−0.05	0.03	−0.32***	0.37***	0.54***	0.57***	0.69
*M*	0.47	10.17	1.61	3.76	4.07	4.18	4.40
SD	0.50	1.69	0.43	0.57	0.53	0.54	0.82
Min	0	7	1	1	1.20	1	1
Max	1	15	3	5	5	5	6
Skewness		−0.23	0.18	−0.44	−0.19	−0.64	−0.42
Kurtosis		−0.74	−0.58	1.76	0.46	1.40	0.67

SASF, social anxiety - fear of negative evaluation; SC, sport commitment; PRS, personal resilience; CRRS, caregiver resilience.

*p < .05;

**p < .01,

***p < .001. Diagonals are squared root of Average variance extracted (AVE) for the five measures. Gender: 1, female; 0, male.

The high correlation between PRS and CRRS (*r* = 0.78) is theoretically plausible, as both dimensions reflect common underlying coping adaptation mechanisms. Previous literature has reported similar high correlations, e.g., *r* = 0.79 in Jonkman’s et al. (2022), *r* = 0.90 in Artuch-Garde’s et al. (2022), and *r* = 0.76 in Van Rensburg’s et al. (2019). Therefore, we considered them as inter-related yet distinct constructs. Also, we allowed the parallel mediators to covary (arrows not presented) to more accurately reflect the shared variance among the residuals for the mediators.

### 3.3 Descriptive statistics

The descriptive statistics and Pearson correlation among the variables are presented in Table 1. Background variables of gender (female) and age were significantly correlated with some of the model variables, and were therefore included as the control variables in the path model step. The predictor, social anxiety, was significantly and negatively correlated with life satisfaction (*r* = −0.32, *p* < 0.001) as well as with the proposed mediators of sport commitment (*r* = −0.21, *p* < 0.001), personal resilience (*r* = −0.41, *p* < 0.001), and caregiver resilience (*r* = −0.36, *p* < 0.001). In addition, life satisfaction was positively and significantly correlated with sport commitment (*r* = 0.37, *p* < 0.001), personal resilience (*r* = 0.54, *p* < 0.001), and caregiver resilience (*r* = 0.57, *p* < 0.001) with moderate magnitude, suggesting the appropriateness of continuing with the proposed mediation model.

### 3.4 Path model with parallel mediators

**FIGURE 2:**
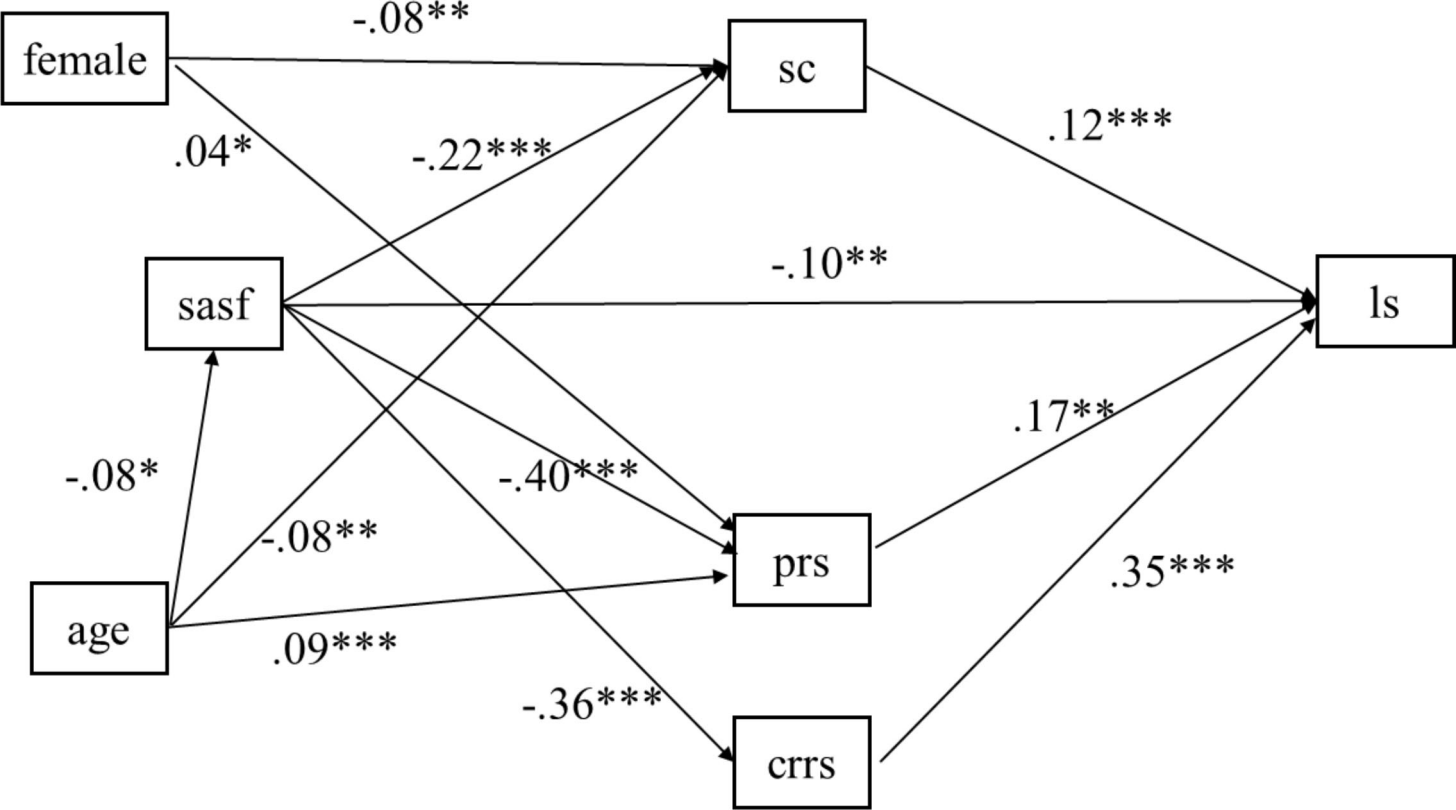
Mediation model with standardized estimates. 1, female; 0, male. *, *p* < 0.05; **, *p* < 0.01; ***, *p* < 0.001.

The path model was specified with sport commitment, personal resilience, and caregiver resilience serving as the mediators in the relationship between social anxiety and life satisfaction, while controlling for the effects of age and gender (female). The standardized estimates are shown in Figure 2. The model demonstrated a satisfactory fit, χ2(5) = 8.414, *p* = 0.135, CFI = 0.998, TLI = 0.992, RMSEA = 0.027, 90% CI = (0.000, 0.057), and SRMR = 0.018. As displayed in Figure 2, social anxiety significantly and negatively predicted sport commitment (β = −0.28, *p* < 0.001), which in turn significantly and positively predicted life satisfaction (β = 0.17, *p* < 0.001). Additionally, social anxiety negatively predicted personal resilience (β = −0.50, *p* < 0.001), which in turn positively predicted life satisfaction (β = 0.26, *p* < 0.01). Moreover, social anxiety negatively predicted caregiver resilience (β = −0.45, *p* < 0.001), which was positively associated life satisfaction (β = 0.53, *p* < 0.001). After controlling for the indirect paths, social anxiety also had significant and negative associated on life satisfaction (*β* = −0.20, *p* < 0.01). Regarding the associations of background variables, it was observed that gender (female vs. male) had a significant negative association on sport commitment and positive association on personal resilience. Age had negative association on social anxiety and sport commitment, and positive impact on personal resilience.

The indirect effects were tested using the bootstrap method with 5,000 bootstrapping samples, and 95% bias-corrected confidence interval was used for significance testing. As shown in Table 2 and Supplementary Table 1, we examined the indirect effects of each mediator in the relation between social anxiety and life satisfaction. All the three mediators of sport commitment [β = −0.05, 95% CI = (−0.08, −0.02)], personal resilience [β = −0.13, 95% CI = (−0.21, −0.06)], and caregiver resilience [β = −0.24, 95% CI = (−0.32, −0.17)] significantly mediated the relationship of social anxiety to life satisfaction. The total indirect effect was −0.41, accounting for 67.93% of the total effect, highlighting the remarkable contribution of the mediators. Moreover, the *R*^2^-value for the four endogenous model variables was 0.364 for life satisfaction, 0.177 for personal resilience, 0.128 for caregiver resilience, and 0.056 for sport commitment, indicating that the 36.4%, 17.7%, 12.8%, and 5.6% of the variations in life satisfaction, personal resilience, caregiver resilience, and sport commitment, respectively, could be explained by the structural model. Additionally, the *R*^2^-value for life satisfaction indicated a large effect size, for personal resilience illustrated a medium effect size, and for caregiver resilience and sport commitment illustrated a small effect size, respectively (Cohen, 1988).

**TABLE 2:** Standardized estimates of total and indirect effects.

IV	Mediator	DV	Estimate	SE	% effect	*P*	Bias-corrected 95% CI
SASF	→SC	→LS	−0.03	0.01	8.15	0.001	[−0.04, −0.01]
SASF	→PRS	→LS	−0.07	0.02	20.69	0.001	[−0.11, −0.03]
SASF	→CRRS	→LS	−0.13	0.02	39.18	< 0.001	[−0.16, −0.09]
SASF	–	→LS	−0.10	0.03	31.97	0.001	[−0.16, −0.04]
Total indirect effect			−0.22	0.02	68.03	< 0.001	[−0.26, −0.18]
Total effect			−0.32	0.03	–	< 0.001	[−0.38, −0.25]

## 4 Discussion

This study aimed to investigate the relationship among social anxiety, life satisfaction, resilience, and sport commitment while accounting for demographic variables of gender and age among primary school students in China. The results revealed that the proposed path model had satisfactory model fit. First, significant gender and age differences were observed among the variables of sport commitment, personal resilience, and social anxiety. Second, positive correlations were found between sport commitment, personal resilience, caregiver resilience, and life satisfaction, while negative associations were observed between these variables and social anxiety, supporting H1 and H2. Third, sport commitment, personal resilience, and caregiver resilience were found to mediate the relationship between social anxiety and life satisfaction, supporting H3 and H4. Our results provide empirical evidence indicating that sport commitment and resilience are crucial factors for enhancing individual and public health. Moreover, individual differences, such as gender and age, should be considered when developing strategies to promote sport commitment and resilience.

Our findings revealed significant gender differences in the measured variables of sport commitment and personal resilience among primary school students in China. The result aligned with previous studies (Tian and Shi, 2022; Zhang Z. et al., 2024), which similarly identified significant gender differences in sport commitment, with boys demonstrating higher levels than girls. The observed gender discrepancy might be attributed to boys’ inclination to engage in more autonomous, consistent, and regular sports activities during their leisure time, driven by a heightened enthusiasm for sports, which ultimately fostered lifelong engagement in physical activities (He et al., 2022). In contrast, girls’ sport commitment might be impacted by insufficient social support from family and peers, as well as traditional social norms and cultural expectations that encourage girls to be more reserved and prioritize education over maintaining regular sports participation (Zhang Z. et al., 2024). Moreover, the findings were consistent with previous research (Jefferies et al., 2018; Qi and Yang, 2024), which identified gender differences in personal resilience, with females demonstrating higher levels than males. This variation might stem from inherent gender distinctions or societal role expectations (Jefferies et al., 2018). Furthermore, girls’ proficiency in establishing interpersonal relationships, particularly their ability to form strong emotional ties with those around them, could contribute to their elevated resilience levels (Qi and Yang, 2024). Additionally, the rapid progression of adolescents’ personal resilience, along with their anticipation of future societal roles, emphasized the critical role of education and social support in nurturing this resilience (Guo and Liang, 2023). To address these disparities, it is imperative to implement targeted interventions that enhance social support while ensuring that these interventions are tailored to meet the unique needs and challenges faced by boys and girls in promoting their overall development.

Our study indicated that both personal and caregiver resilience mediates the relationship between social anxiety and life satisfaction among primary school students in China. These forms of resilience might mediate the negative impact of social anxiety on life satisfaction by providing emotional and psychological support. Furthermore, our findings revealed a positive relationship between personal and caregiver resilience, underscoring the importance of supportive caregiver relationships in helping students cope with adverse life experiences (Qi and Yang, 2024; Wang Y. et al., 2024). Caregiver resilience enables individuals to benefit from family support, which in turn bolsters their personal resilience (Chen et al., 2021). Consistent with previous studies (Shi et al., 2015; Wang and Yao, 2020), our research demonstrated that primary school students with high levels of both personal and caregiver resilience experience greater life satisfaction and lower social anxiety. Elevated resilience levels are often associated with positive attitudes, such as optimism, which help primary school students cope with life challenges and achieve higher life satisfaction (Shi et al., 2015). A stable and healthy caregiver relationship fosters a nurturing environment that supports effective management of life’s challenges, especially during early childhood and adolescence. This support is crucial for children and adolescents, who rely on social and emotional resources to navigate developmental changes and challenges (Pinkerton and Dolan, 2007), particularly those from under-resourced families and communities (Marlotte et al., 2023). Proactive strategies by individuals and families can significantly mitigate the impact of adverse situations (Walsh, 2016). Enhanced resilience in both individuals and caregivers facilitate the development of critical cognitive and communicative skills necessary for navigating traumatic experiences and building effective support systems (Saltzman, 2016). These support systems often develop through successful resolution of conflicts and enhancement of communication skills (Kalisch et al., 2017). Moreover, life satisfaction may be more influenced by how students’ interpretations of events rather than the events themselves (Yang et al., 2024). By fostering personal and caregiver resilience, individuals can redirect their focus away from negative perceptions of social interactions, thereby improving relationships with their surroundings and alleviating social anxiety (Jefferies et al., 2021). Therefore, resilience functions as a protective factor, diminishing the fear of negative evaluation and enhancing life satisfaction.

The research demonstrated that sport commitment serves as a mediator between social anxiety and life satisfaction among primary school students in China. The findings indicated that sport commitment was associated with the social anxiety and life satisfaction within this sample. As a result, primary school students who exhibited higher levels of sport commitment reported enhanced life satisfaction and diminished social anxiety. These findings were consistent with previous studies (Dong et al., 2024; Pulido et al., 2018; Murillo et al., 2022), which have consistently demonstrated a positive relationship between sport commitment and health-related factors. Moreover, the sport commitment framework highlighted the significant role of psychological commitment in sustaining students’ involvement in sports. This framework theorized that psychological commitment is reflected in an individual’s persistent desire to continue participating in sport, a desire significantly influenced by the satisfaction and enjoyment derived from the activity (Scanlan et al., 1993a). A core idea of this framework is the notion that satisfaction is a critical determinant of sport commitment. Satisfaction and enjoyment result from intrinsic motivation, which is categorized into three distinct types: pleasure derived from acquiring knowledge, fulfillment from skill development or creation, and emotional gratification through active engagement (Zahariadis et al., 2006). According to the Sport Commitment Theory, satisfaction is not merely a byproduct of participation but an essential element that actively influences an individual’s ongoing commitment to sport (Carpenter and Scanlan, 1998). Building on this, our findings suggested that sustained engagement in sports prioritizes health benefits, such as alleviating social anxiety and improving life satisfaction and self-fulfillment, consistent with a comprehensive approach to holistic health (Welk et al., 2003). Moreover, despite the implementation of several policies to promote physical fitness and health among Chinese students (Zhang Z. et al., 2024), a declining trend in health-related physical fitness among Chinese college students has been observed (Dong et al., 2023b). In light of these findings, the dedication to lifelong physical activity is crucial for both individual and public health (Williams, 2013). Previous studies indicated a positive relationship between sport commitment and continuous engagement intention (Choi, 2023). Our findings suggested that incorporating sport commitment as a core component in designing interventions could effectively promote a persistent engagement in physical activity by fostering individuals’ motivation and, consequently, enhancing health-related fitness and psychological well-being.

### 4.1 Implications

The findings of this study present significant theoretical and practical implications. Theoretically, the study enhances our understanding by elucidating two critical aspects: first, the mediating role of personal and caregiver resilience in the relationship between social anxiety and life satisfaction; second, the pivotal role of sport commitment in mediating the link between social anxiety and life satisfaction. These insights deepen our theoretical comprehension of wellbeing among primary school students. Practically, the study highlights the importance of developing school-based and community-based programs that enhance both personal and caregiver resilience. Specifically, schools could integrate structured sport activities and resilience training sessions that help students develop coping skills, alleviate social anxiety, and strengthen sport commitment. Integrating sport commitment as a core element, such programs may be beneficial for young students, as consistent and enjoyable participation in sports can reduce social anxiety and promote life satisfaction. Additionally, the study advocates for a holistic approach to students’ wellbeing, that encompasses physical, emotional, and psychological dimensions. Educational institutions should adopt comprehensive strategies that incorporate both sport commitment and resilience-building activities into the curricula, thereby facilitating the overall development of students.

### 4.2 Limitations

This study has several limitations that must be acknowledged. First, the cross-sectional design restricts the capacity to establish causal links between the variables. Longitudinal research is required to investigate the directionality of the observed associations. Second, employing a convenience sampling method may limit the generalizability of the findings to other populations, as the sample was obtained from four primary schools in specific Chinese cities. Third, the dependence on self-reported measures could introduce response bias, as participants could have provided socially desirable responses. Future research should consider using multi-informant assessments or objective measures to validate the findings. Finally, although the study controlled for gender and age, it did not account for other potential confounding factors such as socioeconomic status, parental education, and cultural influences, which may have impacted the results.

### 4.3 Conclusion

The present study provides empirical evidence suggesting that sport commitment and resilience are associated with higher life satisfaction and lower levels of social anxiety among Chinese primary school students. These associations should be interpreted cautiously, given the cross-sectional design and reliance on self-report measures, which limit causal inference. Observed variations across gender and age groups underscore the importance of considering development and social contexts when designing future research and interventions. From a practical perspective, the findings suggest that school-based programs that encourage regular physical activities and strengthen resilience may be promising avenues for supporting students’ emotional wellbeing; however, such programs should be rigorously evaluated through longitudinal and controlled studies before large-scale implementation. The study contributes to a more nuanced understanding of the interplay among social anxiety, life satisfaction, resilience, and sport commitment, offering a foundation for future research aimed at informing evidence-based mental health initiatives in primary school settings.

## Data Availability

The raw data supporting the conclusions of this article will be made available by the authors, without undue reservation.
