# Supplementary material for: Exploring the mediating roles of sport commitment and resilience between life satisfaction and social anxiety among Chinese primary school students
Source: Front Psychol. 2025 Nov 5;16:1619817. doi: 10.3389/fpsyg.2025.1619817 (PMC12627039; doi:10.3389/fpsyg.2025.1619817)
Supplement: Supplementary file 1 [file Table_1.docx]

**Supplement**

Supplement 1. Table for unstandardized estimates of total and indirect effects

| IV | Mediator | DV | Estimate | SE | % effect | *p* | Bias-corrected 95% CI |
| --- | --- | --- | --- | --- | --- | --- | --- |
| sasf | → sc | → ls | -0.05 | 0.02 | 8.06 | 0.001 | [-0.08, -0.02] |
| sasf | → prs | → ls | -0.13 | 0.04 | 20.89 | 0.001 | [-0.21, -0.06] |
| sasf | → crrs | → ls | -0.24 | 0.04 | 39.14 | <.001 | [-0.32, -0.17] |
| sasf | | → ls | -0.20 | 0.06 | 32.07 | 0.001 | [-0.30, -0.08] |
| Total indirect effect | |  | -0.41 | 0.04 | 67.93 | <.001 | [-0.49, -0.34] |
| Total effect | | | -0.61 | 0.06 |  | <.001 | [-0.73, -0.49] |
