# Supplementary material for: Exploring the mediating roles of sport commitment and resilience between life satisfaction and social anxiety among Chinese primary school students
Source: Front Psychol. 2025 Nov 5;16:1619817. doi: 10.3389/fpsyg.2025.1619817 (PMC12627039; doi:10.3389/fpsyg.2025.1619817)
Supplement: Supplementary file 2 [file Table_2.docx]

**Supplement**

Supplement 2. Table for comparisons of ML vs WLSMV estimators in terms of CFA factor loadings.

|  | ML | WLSMV |
| --- | --- | --- |
| LS |  |  |
| LS1 | 0.69 | 0.73 |
| LS2 | 0.73 | 0.79 |
| LS3 | 0.78 | 0.86 |
| LS4 | 0.76 | 0.82 |
| LS5 | 0.53 | 0.52 |
| SASF |  |  |
| SA1 | 0.58 | 0.74 |
| SA2 | 0.67 | 0.77 |
| SA3 | 0.77 | 0.83 |
| SA4 | 0.70 | 0.84 |
| SA5 | 0.78 | 0.85 |
| SA6 | 0.79 | 0.89 |
| SC (5 subscales) | |  |
| Commitment | |  |
| SC1 | 0.53 | 0.65 |
| SC2 | 0.82 | 0.87 |
| SC3 | 0.87 | 0.93 |
| Constraints |  |  |
| C1 | 0.69 | 0.73 |
| C2 | 0.84 | 0.77 |
| C3 | 0.79 | 0.93 |
| Opportunity |  |  |
| O1 | 0.68 | 0.73 |
| O2 | 0.84 | 0.89 |
| O3 | 0.83 | 0.91 |
| Enjoyment |  |  |
| SJ1 | 0.88 | 0.95 |
| SJ2 | 0.94 | 0.96 |
| SJ3 | 0.93 | 0.95 |
| Investment |  |  |
| IV1 | 0.65 | 0.69 |
| IV2 | 0.56 | 0.65 |
| IV3 | 0.82 | 0.92 |
| PRS |  |  |
| PRS1 | 0.67 | 0.76 |
| PRS2 | 0.51 | 0.59 |
| PRS3 | 0.63 | 0.70 |
| PRS4 | 0.73 | 0.79 |
| PRS5 | 0.73 | 0.79 |
| PRS6 | 0.77 | 0.83 |
| PRS7 | 0.75 | 0.81 |
| PRS8 | 0.69 | 0.76 |
| PRS9 | 0.72 | 0.79 |
| PRS10 | 0.61 | 0.74 |
| CRRS |  |  |
| CRRS1 | 0.61 | 0.66 |
| CRRS2 | 0.66 | 0.72 |
| CRRS3 | 0.62 | 0.70 |
| CRRS4 | 0.70 | 0.76 |
| CRRS5 | 0.63 | 0.73 |
| CRRS6 | 0.65 | 0.77 |
| CRRS7 | 0.69 | 0.78 |
